# P-578. Just “Short of a Cure”: Unique Considerations in Uptake and Use of Long-Acting Injectable Cabotegravir/Rilpivirine Among Long-Term Survivors with HIV

**DOI:** 10.1093/ofid/ofae631.776

**Published:** 2025-01-29

**Authors:** Priyasha Pareek, Lauren F Collins, Kaylin Dance, Mollie Smith, Alicia Dawdani, Xavier A Erguera, Ryan Walker, Janet Grochowski, Francis Mayorga-Munoz, Matt Hickey, Mallory Johnson, John Sauceda, Jose I Gutierrez, Laurence Balter, Moira McNulty, Kimberly Koester, Katerina Christopoulos, Jonathan Colasanti

**Affiliations:** Emory University School of Medicine, Atlanta, Georgia; Emory University School of Medicine, Division of Infectious Diseases, Atlanta, Georgia; Emory University, Atlanta, Georgia; University of Massachusetts Chan Medical School, Essex, Massachusetts; University of Chicago, Chicago, Illinois; University of California, San Francisco, San Francisco, California; Columbia University, New York, New York; University of California, San Francisco, San Francisco, California; UCSF, Concord, California; University of California, San Francisco, San Francisco, California; University of California, San Francisco, San Francisco, California; University of California San Francisco, San Francisco, California; University of California, San Francisco, San Francisco, California; Emory University, Atlanta, Georgia; University of Chicago, Chicago, Illinois; University of California, San Francisco, San Francisco, California; University of California San Francisco, San Francisco, California; EMORY UNIVERSITY SCHOOL OF MEDICINE, Atlanta, GA

## Abstract

**Background:**

Understanding perspectives of subgroups of people with HIV (PWH) who have accessed long-acting cabotegravir/rilpivirine (CAB/RPV-LA) is crucial for optimizing implementation; especially among long-term survivors (LTS) who have lived through the trauma of the early epidemic to the innovation of today.

**Methods:**

We analyzed semi-structured interviews collected from 08/2022-05/2023 via a parent study at four Ryan White clinics in Atlanta, Chicago, and San Francisco among early adopters of CAB/RPV-LA (received ≥3 injections or discontinued). Our analysis explored factors impacting CAB/RPV-LA uptake and use in LTS (living with HIV since 2000 or earlier) using a socioecological model of HIV care engagement.

**Results:**

Among 20 LTS, median age was 56 (Q1-Q3 54-60) years and median time living with HIV was 27 (Q1-Q3 25-34) years. Thirteen (65%) identified as racial/ethnic minorities and seven (35%) as cis or trans women. We identified three primary themes: 1) The majority of LTS eagerly awaited CAB/RPV-LA and seemed unconflicted in their choice to switch from oral antiretroviral therapy (ART). Their confidence stemmed from experience using new iterations of oral ART over decades, with some citing CAB/RPV-LA as being a step closer to a cure. 2) Choosing to start CAB/RPV-LA was sometimes facilitated by input from longstanding, trusted relationships with healthcare providers or other PWH. 3) Reflections on the emotional gravity of witnessing loved ones die from AIDS-related illnesses early in the epidemic, and the mental imprisonment of around-the-clock pill taking required for their own survival, influenced use of CAB/RPV-LA. In fact, many reported transforming HIV-related psychological trauma and tragedy into education, support, and advocacy efforts: using CAB/RPV-LA was a unique mechanism to further benefit their community—indeed, it was an obligation to both PWH who have passed as well as PWH to come, who could learn from their experience.

**Conclusion:**

The perspectives of LTS who initiated CAB/RPV-LA during early implementation revealed uptake and use was shaped by the emotional, psychosocial, and physical impact of living with HIV for decades. These data serve as a framework to tailor service delivery of current and future long-acting ART options in this population.

**Disclosures:**

**Moira McNulty, MD**, Gilead Sciences, Inc.: Advisor/Consultant **Katerina Christopoulos, MD, MPH**, Janssen: Honoraria

